# Examining the Attachment of Artificial Teeth to the Acrylic Base Through Several Adhesions and Polymerizing Techniques

**DOI:** 10.1155/ijod/7256608

**Published:** 2026-01-05

**Authors:** Ibrahim H. Alfahdawi

**Affiliations:** ^1^ Department of Prosthodontics, College of Dentistry, University of Al-Amaarif, Ramadi, Al-Anbar, Iraq, bamdc.edu.pk

**Keywords:** acrylic denture base, acrylic denture teeth, microwave, shear bond strength, surface treatment

## Abstract

**Background:**

The strength of the binding between the acrylic denture teeth and the foundation materials of dentures has been found to vary.

**Objectives:**

Analyze the various adhesives and polymerizing techniques used to attach acrylic denture teeth to the acrylic denture base.

**Methods:**

Tooth preparation and curing were done using heat‐cured and microwave‐cured (Acron Mc) acrylic resin. The 60 teeth were divided into six groups and subjected to different surface treatments. The second group was given diatoric preparation, the third group thinner, the fourth group acetone, the fifth group diatoric acetone conditioning, and the sixth group diatoric thinner conditioning. The control group was left untreated. The mesiodistal retention grooves of the second, fifth, and sixth groups were 2 mm deep and 3 mm wide, significantly affecting (*p*  < 0.01) the ridge laps of the acrylic denture teeth. The specimens were stored for 10 days at 37°C in distilled water before testing. The specimen was then visually evaluated after being broken by shear force delivered by an Instron universal testing machine.

**Results:**

The study revealed that the microwave‐cured acrylic denture base displayed higher shear bond strength (SBS) compared to the heat‐cured resin, regardless of the surface treatment applied. The outcomes indicated that the microwave‐cured resin had notably lower mean values of SBS for all acrylic denture teeth in comparison to the heat‐cured resin.

**Conclusions:**

The microwave‐cured resin had significantly lower mean SBS values than the heat‐cured resin. The sixth group had the largest SBS compared to the other groups at the *p*‐value < 0.05. Unlike previous studies, this work incorporates a comparative evaluation of combined mechanical and chemical treatments (grooves with thinner conditioning and acetone), aiming to generate clinically applicable modifications for enhancing denture longevity.

## 1. Introduction

The loss of natural teeth is a frequent issue that needs serious consideration because the majority of individuals need to replace their lost teeth. Artificial dentures are the most preferred treatment option for older adults since they are less costly than implants and fixed prostheses. Because of their chemical connection to the denture base material, acrylic teeth are usually preferred over porcelain teeth since they are easily accessible, reasonably priced, and swiftly cut [1].

In recent times, there has been an increasing interest in enhancing the adhesive strength between artificial teeth and denture bases. This is essential for maintaining the durability and steadiness of dentures, as a poor bond can result in frequent maintenance and renewals. Hence, scientists have studied various methods of surface treatment and polymerization to improve the bonding strength between artificial teeth and denture bases. Polymethyl methacrylate (PMMA) is commonly used to make denture bases due to its cost‐effectiveness, ease of use, esthetic appeal, and dimensional stability [2]. The strength of the bond between the denture base and teeth plays a crucial role in the durability of removable dentures. Shear bond strength (SBS) analysis is the most widely used testing method to assess the bonding strength between artificial teeth and denture base resins [3, 4]. The most common clinical issue that requires repair is the debonding of a tooth from a complete or partial removable denture base [5].

The connection between the denture teeth and base material occurs during processing, as the teeth are securely fixed in the base and polymerized together [6, 7]. Even though this bond is strong, there is still a risk of the denture teeth becoming detached due to different reasons [8]. These reasons may involve ineffective bonding methods used in denture creation, insufficient polymerization of the base material, or excessive pressure on the denture while in use [9].

The strength of the binding between the prosthetic teeth and the foundation materials of dentures has been found to vary [10]. The loss of natural teeth is a frequent issue that needs serious consideration because the majority of individuals need to replace their lost teeth. Artificial dentures are the most preferred treatment option for older adults since they are less costly than implants and fixed prostheses. Because of their chemical connection to the denture base material, acrylic teeth are usually preferred over porcelain teeth since they are easily accessible, reasonably priced, and swiftly cut [11].

Introduction of crosslinked acrylic teeth led to an increase in their resistance to stain, fracture, and abrasion, however, it also resulted in a decrease in their bond strength with the denture base [12]. Reattaching acrylic teeth to the denture base, either through mechanical or chemical means, poses a challenge for both dentists and patients. Loose teeth, predominantly in the anterior region, were found to be the cause of a significant percentage of denture repairs, ranging between 22% and 30%. This issue is attributed to the limited ridge lap surface available for bonding and the specific stresses encountered during daily activities [13].

The mechanical method, such as roughening the surface with sandblasting, is time‐consuming and is said to enhance bond strength [14]. Chemicals, like monomers, nonpolymerizable solvents, dissolved PMMA, and various mixtures or adhesives, are utilized in chemical procedures [15]. The chemical makeup of PMMA plays a role in the connection between the denture base and the tooth [16]. The alignment of functional forces and the available space for bonding with the denture base (ridge lap area) are two potential reasons for teeth becoming detached from the acrylic denture base. The primary reason for denture teeth detachment from wax or tin foil contamination is believed to be the main cause of adhesive failure [17, 18].

Microwave technology has many benefits and uses in the field of prosthetic dentistry. The microwave technique resulted in a significantly shorter curing time, a shorter dough‐forming time, more homogenous dough, fewer acrylic resin color changes, fewer fractures of the resin base and artificial teeth, and a simple, clean process [1, 14]. A light plastic flask was used in place of the heavy brass flask, and a microwave was used in place of the water bath tank [19].

The purpose of this research is to assess the SBS between acrylic teeth and the denture base by applying several treatment materials (diatoric preparation, acetone [dimethyl ketone], and thinner [turpentine]) and utilizing both conventional and microwave curing methods.

## 2. Materials and Methods

To guarantee uniformity in the materials used for denture bases and artificial teeth (florident, cross‐linked acrylic teeth), 60 examples were carefully chosen. Six groups were created from these specimens, with five specimens in each group as follows:

The first group is teeth that have not received any surface modification (control).
Second group: Diatoric preparation (retention groove) is used to condition teeth.
Third group: Thinner teeth without groove.
Fourth group: Acetone‐conditioned teeth without groove.
Fifth group: Teeth with retention grooves are conditioned with acetone.
Sixth group: Teeth with a retention groove that have been conditioned with thinners.

**Figure 1:**
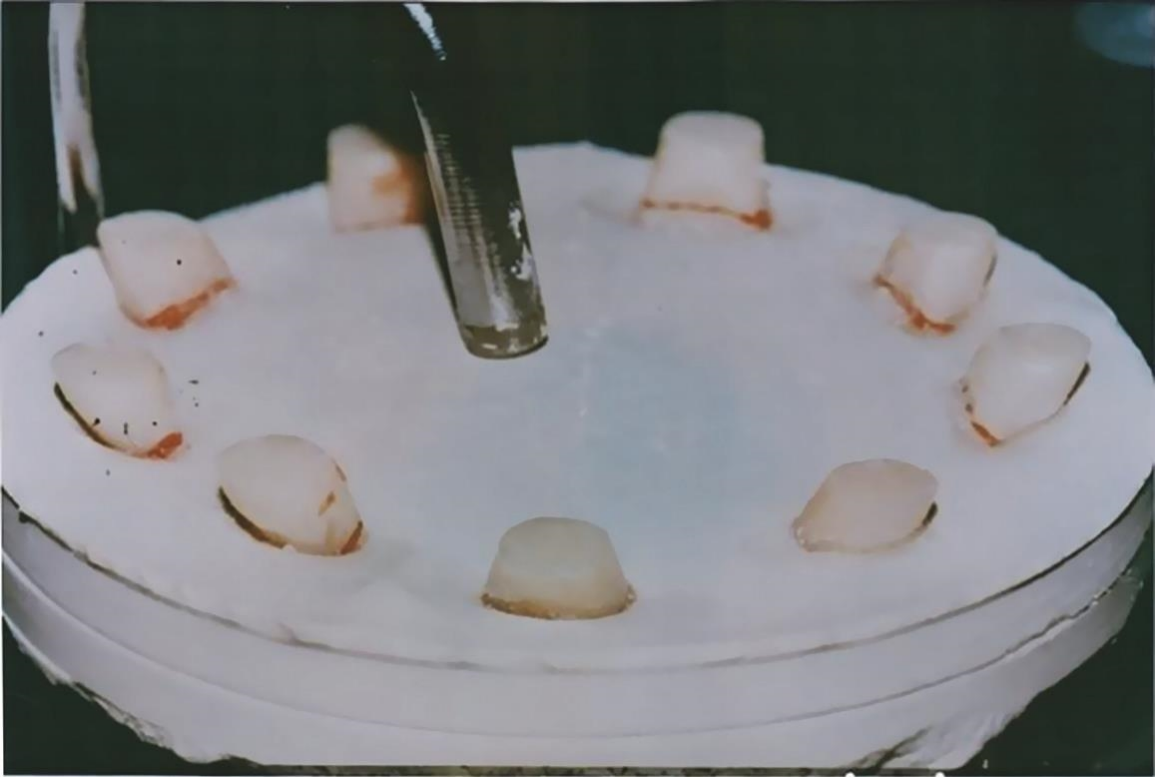
Acrylic central incisors.

Each group’s specimens were treated by using microwave and heat‐cured techniques. The following describes the 60 maxillary acrylic central incisors (left and right), which were all cut at the gingival part of the neck and came from the same mold in terms of size and shape (Figure 1). The circular stone base shape, measuring 5 mm in diameter and 13 mm in thickness, was prepared by using a rubber casting mold. The manufacturer’s directions were followed when mixing and pouring the dental stone into the rubber mold. To align the gingival portion of the tooth with the horizontal plane, approximately 4 mm of each central incisor’s section that had previously been measured and marked was embedded in the stone‐thick mixture. Then, the teeth were removed from the stone base to be ready for fixation on the wax block [20]. An inverted cone carbide bur controlled by a stopper was used to prepare the retention groove on the ridge lap surface of the artificial acrylic teeth, creating a groove 3 mm wide and 2 mm depth at the bottom (measured by using the Vernier caliper). For the second, fifth, and sixth groups, the groove expanded 5 ± 0.5 mm mesiodistally.

**Figure 2:**
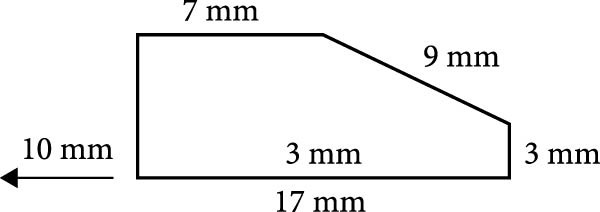
The rectangular wax block’s metal mold.

A rectangular depression was present in a brass metal mold. This hollow measures 17, 7, 9, and 3 mm in diameter, with a 3 mm hollow within (Figure 2) [21].

**Figure 3:**
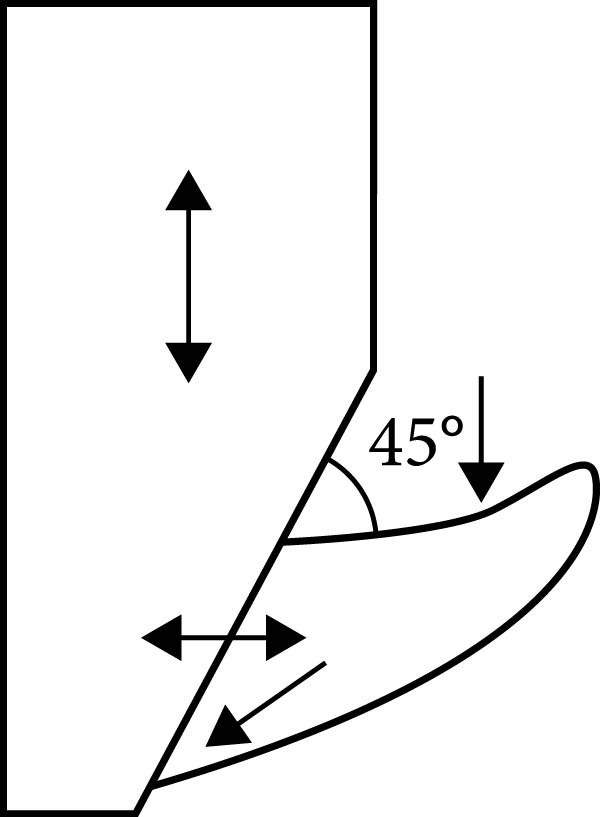
Alignment of tooth on the beveled surface.

The wax was poured into the metal mold after it had melted in a small stainless steel container, which was used only for wax heating. When the wax solidified and the wax block was removed, the teeth were waxed with a beveled surface on the rectangular wax block. Every denture tooth was aligned by the beveled surface’s slope so that the long axis of each tooth could be measured with a Vernier at a 45‐degree angle from the base of the wax block (its use in this study was solely for specimen alignment in acrylic resin processing) (Figure 3).

**Figure 4:**
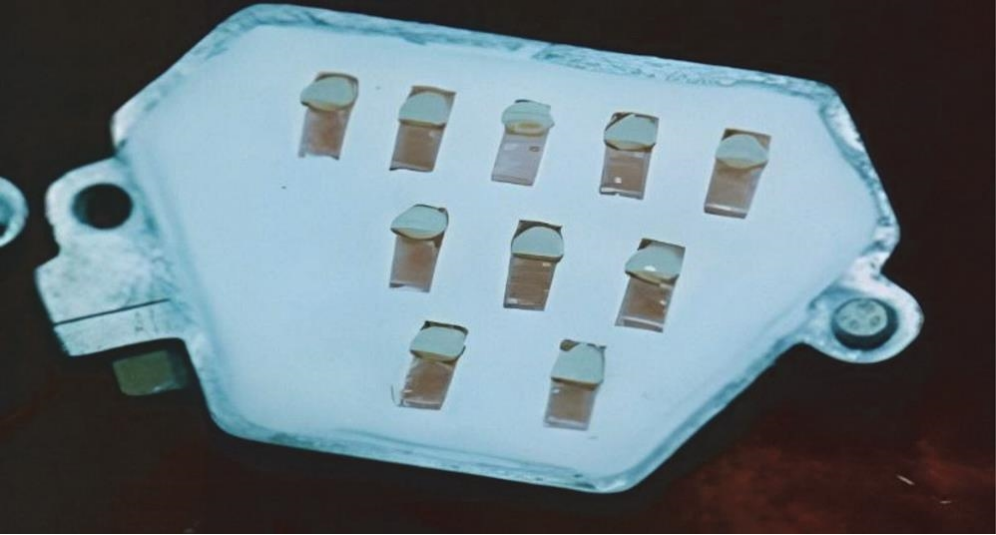
Samples set within the metal mold and the flask.

The necessary teeth were placed into the mold after the traditional flasking procedure for a complete denture. The mold was prepared using the standard flasking method for full dentures. To prepare for packing with acrylic dough (PMMA), both halves of the flask were uniformly coated with a cellulose‐based separating medium (aKriLEKS) and allowed to air dry. This step prevented the acrylic resin from adhering to the metal flask during polymerization (Figure 4). After that, the mold was filled with the proper teeth.

The acrylic resin (Acron Mc) was mixed using the manufacturer’s recommended powder‐to‐liquid ratio, manipulated until the late dough stage, and cured in cycles. Polymerized following the specified curing protocols: heat polymerization (74°C for 2 h + 100°C for 1 h) or microwave polymerization (3 min at 650 W). These procedures were followed precisely to ensure consistency across all experimental samples.

The acrylic resin was packed during the late dough stage, which was confirmed by its clean separation from the walls of the glass mixing jar. The acrylic resin dough was added after the tooth samples were placed into the mold and exposed to acetone or thinner for 180 s. Then, the two halves were combined and placed under the press. The pressure was then progressively increased to let the dough spread uniformly over the mold before it was released.

**Figure 5:**
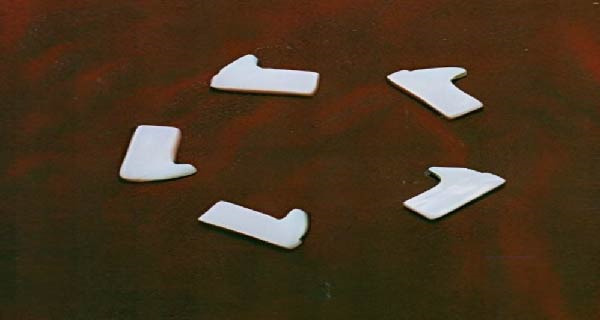
Specimens are prepared for testing after being polished and finished.

After meticulously de‐flasking and cleaning each sample, acrylic burs were used to remove any remaining flashes (Figure 5). After meticulously de‐flasking and cleaning each sample, acrylic burs were used to remove any remaining flashes (Figure 5). After deflasking, specimens were finished using a stone bur and 120‐grain sandpaper under water cooling to ensure surface smoothness. These steps were performed as part of routine mechanical finishing and did not involve any refractory processes. The samples were continuously cooled with water to ensure uniform size, as verified by a Vernier.

**Figure 6:**
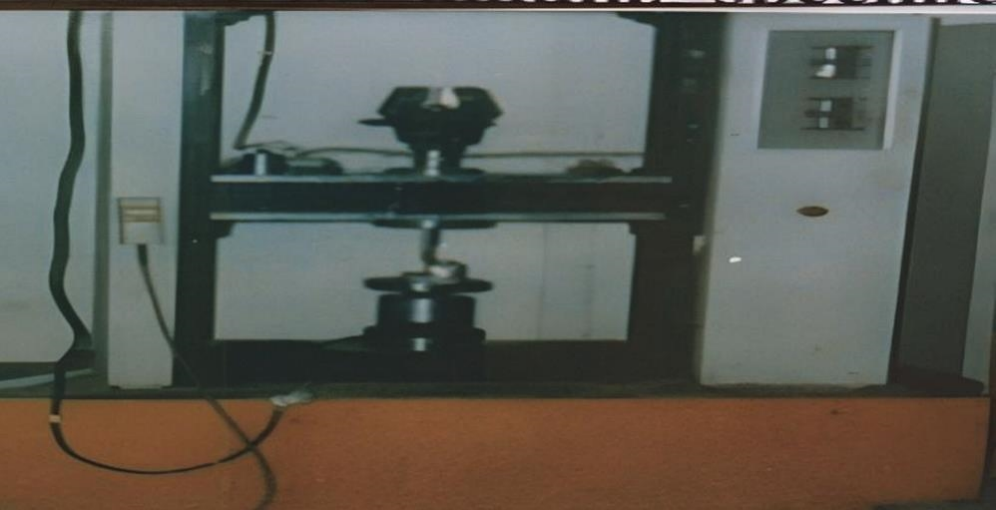
The Instron universal testing machine.

The model 1195 (Instron Corporation, Canton, Mass) was an Instron Universal Testing Machine. A 1000 N load cell and a cross‐head speed of 0.5 mm/min (Figure 6) were used. Using Newton’s Instron graph reader, specimens were loaded until they fractured, and the load was recorded [14]. Based on the (*F*) in (N) at fracture and the adhesive surface area (*S*) in (mm^2^), the SBS was computed and translated to (MPa).

B.S.=FS    S=π4D2.

*D* (diameter) = 5 mm, *S* = 19.64 mm^2^, B. S. = bond strength (N/mm^2^), *F* = force at failure (N), and *S* = area of cross‐section [22]. The fracture sites were evaluated by visual examination.

### 2.1. Statistical Analysis

All statistical analyses were performed using IBM SPSS Statistics for Windows, Version 25.0 (IBM Corp., Armonk, NY, USA). The Shapiro–Wilk test was applied to verify the normality of the data distribution, and Levene’s test was used to confirm the homogeneity of variances (heteroscedasticity).

When statistically significant differences were detected (*p*  < 0.05), Tukey’s honest significant difference (HSD) post hoc test was applied to identify which groups differed significantly. Data are presented as mean ± standard deviation (SD). For clarity, tables and figures include grouping letters: lowercase letters indicate significant differences among heat‐cured groups, while uppercase letters indicate differences among microwave‐cured groups.

Instead of using multiple separate analyses (one‐way ANOVA and *t*‐tests), a two‐way ANOVA was employed to simultaneously evaluate the effects of curing method and surface treatment, as well as their interaction, on SBS. This single comprehensive analysis improves clarity and statistical robustness. Tukey’s HSD post hoc test was subsequently applied to identify which groups differed significantly.

## 3. Results

**Figure 7:**
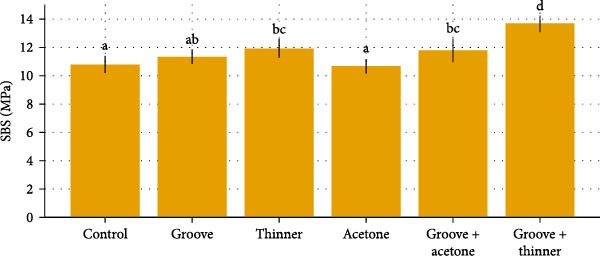
Heat‐cured groups: mean ± SD with significance letters.

**Figure 8:**
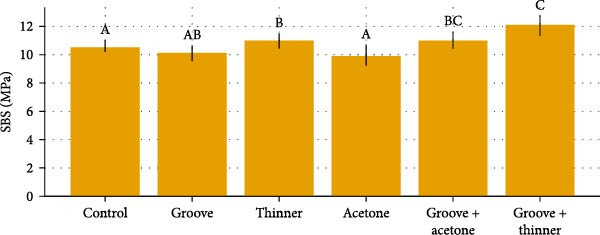
Microwave‐cured groups: mean groups ± SD with significance letters.

**Figure 9:**
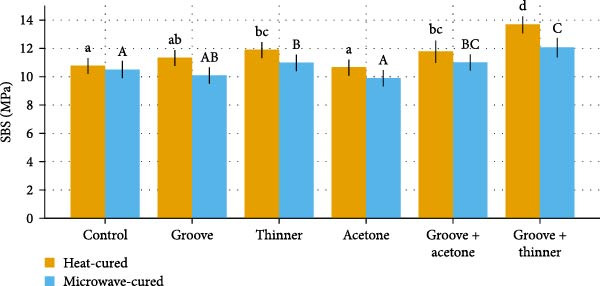
Heat vs. microwaves: mean ± SD with significance letters.

A total of 120 specimens were tested (*n* = 10 per treatment × six surface treatments × two curing methods). Descriptive results (mean ± SD) and significance groupings are summarized in the comparative Table 1, and updated Figures 7–9 visualize group trends with statistical grouping letters (lowercase for heat‑cured, uppercase for microwave‑cured).

**Table 1:** Shear bond strength (SBS, MPa) of acrylic resin groups.

Acrylic resin/SBS	Control	Groove	Thinner	Acetone	Groove + acetone	Groove + thinner
Heat‐cured	10.78 ± 0.60^a^	11.35 ± 0.50^ab^	11.92 ± 0.71^bc^	10.68 ± 0.52^a^	11.80 ± 0.82^bc^	13.68 ± 0.59 days
Microwave‐cured	10.47 ± 0.55^A^	10.08 ± 0.55^AB^	10.98 ± 0.52^B^	9.91 ± 0.74^A^	11.00 ± 0.59^BC^	12.06 ± 0.73^C^

*Note:* Horizontally, different superscript uppercase letters indicate significant differences within microwave‐cured groups; vertically, different superscript lowercase letters indicate significant differences within heat‐cured groups (Tukey test, *p*  < 0.05).

A two‑way ANOVA was performed with curing method (heat vs. microwave) and surface treatment (control, groove, thinner, acetone, groove + acetone, and groove + thinner) as fixed factors and SBS (MPa) as the dependent variable. The analysis showed significant main effects of curing method (*p*  < 0.001) and surface treatment (*p*  < 0.001), as well as a significant curing method × surface treatment interaction (*p*  < 0.01), indicating that the effect of surface treatment differed depending on the curing modality.

### 3.1. Heat‑Cured Groups

Within heat‑cured acrylic, SBS differed by surface treatment (Tukey HSD post hoc following the two‑way ANOVA). The groove + thinner group achieved the highest SBS (13.676 ± 0.586 MPa; letter “d”), exceeding control and acetone (both “a”; *p*  < 0.001) and outperforming groove and thinner (letters “ab” and “bc”, respectively). Acetone produced the lowest SBS (10.680 ± 0.517 MPa). These patterns are displayed in Figure 7 with lowercase letters over each bar to denote groups that differ significantly (different letters = *p*  < 0.05).

### 3.2. Microwave‑Cured Groups

For microwave‑cured acrylic, the groove + thinner treatment also produced the highest SBS (12.061 ± 0.729 MPa; letter “C”), whereas acetone yielded the lowest SBS (9.919 ± 0.744 MPa; letter “A”). Post hoc comparisons indicated significant differences for groove + thinner versus control and versus acetone (both *p*  < 0.001), and additional contrasts consistent with the assigned uppercase letters (Figure 8).

### 3.3. Comparison Between Curing Methods

Across all surface treatments, heat‑cured specimens exhibited higher SBS than microwave‑cured specimens, consistent with the significant main effect of curing method. Simple‑effects contrasts (Tukey HSD within the two‑way model) confirmed that the heat‑cured SBS exceeded the corresponding microwave‑cured SBS for the same surface treatment in multiple cases, with the largest absolute advantage observed for groove + thinner. Figure 9 presents grouped bars (heat vs. microwave) for each surface treatment with both lowercase and uppercase letters to summarize within‑method differences.

## 4. Discussion

Numerous investigations have been conducted to assess and enhance acrylic teeth’ adhesiveness to dentures’ base material. Numerous factors, such as the materials’ cross‐linking and the availability of solvents, might influence bond strength and how contaminated the procedure was. This study used a shear test to assess the binding strength between two denture base resins and artificial acrylic teeth. Both descriptive and inferential statistical analyses were performed on the results. A significant threshold of (*p*  < 0.05) and (*p*  < 0.001) was set. Every value was given in millipascals.

The study’s findings indicate that, about various surface treatments, the denture base resin’s surface bond strength of acrylic teeth cured in heat‐cured was substantially (*p*‐value < 0.05) higher than that of denture base resin cured in a microwave. The acrylic resin and polymerization process influenced the tooth‐to‐denture base bond strength, as seen by the decreased SBS observed in the microwave‐cured resin groups compared to the heat‐cured resin groups.

This agrees with results obtained by Takahashi et al. [20] and Schneider et al. [21], suggesting that during the microwave method, compared to the usual water‐bath procedure, the unpolymerized base material was in contact with the tooth surface for a much shorter amount of time. Furthermore, the reduced interpenetration of the denture base polymer network and the tooth was seen due to the nature of the acrylic resin that was microwave‐cured. The reduced interpenetration of the denture base polymer network and the tooth could lead to decreased bonding strength between the two materials. In addition, the shorter contact time between the unpolymerized base material and the tooth surface may affect the overall adhesion and durability of the denture.

The novelty of this study lies in the simultaneous evaluation of multiple conditioning methods under both microwave and heat polymerization cycles. While earlier reports examined these factors separately, our approach reflects real‐world denture fabrication practices where clinicians often combine mechanical grooves with chemical solvents to boost retention. This combined approach offers incremental yet clinically meaningful innovation, directly applicable to everyday prosthodontic procedures. Unlike previous research, this work shows that combining groove preparation with thinner conditioning produces a synergistic effect, significantly enhancing bond strength beyond either method alone. This integrated approach represents a practical, chairside strategy that can help extend the longevity of prostheses.

Less swelling could be the cause, resulting in less penetration, weakening the relationship. The precise degree of cross‐linking in resins cured in a water bath or microwave was uncertain. When denture base resin was first produced, it tended to craze and fracture readily; this was helped by cross‐linking, previously studied by Al‐Dwairi et al. [22].

However, mode bonding to dental plastic was more challenging to obtain by Batisse and Nicolas [23]. The microwave‐cured resin could have many cross‐links. Only some functional groups have been available for bonding since there was less unlinked polymer. As for this, adhesive bond failure and poor SBS values were the main characteristics of microwave‐cured resin. In agreement with Al‐Somaiday et al. [24] and Abu‐Anzeh [25], the results of all surface treatments showed a significant difference (*p*‐value <0.05) in enhancing the SBS mean value. Since thinner is made up of multiple solvents, it is a stronger solvent than acetone and can dissolve polymers more completely. As a result, teeth treated with thinner failed in cohesive mode, so this improved polymer network could enhance the mechanical strength and durability of the denture base. The solvents may also have contributed to a more homogeneous distribution of the polymerizable monomer, resulting in a more uniform and stable denture material, this finding is in agreement with Hariharan et al. [26].

In addition to creating a smoother surface for the denture base resin to adhere to, this resulted in a more secure and durable bond between the denture teeth and the base, improving the overall stability and longevity of the denture as previously studied by Khanna et al. [27]. Thus, the monomer‐to‐acrylic resin penetration and polymerization along the bonding surface of grooved teeth treated with thinner failed mostly in the cohesive mode, encouraging chemical and mechanical interlocking. Acetone is a powerful solvent for PMMA, effectively removing most micro debris and smoothing the adhesive surface, producing a structure resembling a sponge, in agreement with Vergani et al. [28]. According to Al‐Ani [29], adding acetone to the bond surface increases the mobility of the denture base’s monomer units, increasing the number of active sites and causing a physical interaction (Vander Waals forces). Although these forces result in a strong bond, no true chemical cross‐linking reaction exists. The acetone surface treatment will produce a lower mean bond strength value than the polymerization reaction between the two curing techniques with a thinner surface treatment. A strong solvent for PMMA, acetone, can be used to wet the surface. It removes most micro debris, smooths the adhesive surface, and forms a sponge‐like structure. This increases bond strength through a physical interaction known as Vander Waals forces.

The clinical relevance of these findings is clear. Enhanced SBS between artificial teeth and the denture base directly translates into fewer incidences of tooth debonding, one of the most common causes of denture repair. In particular, the groove + thinner combination demonstrated significantly higher bond strength, suggesting that routine incorporation of simple chairside mechanical and solvent‐based pretreatments could extend prosthesis service life, reduce repair costs, and improve patient satisfaction. Furthermore, the study underscores that although microwave curing offers efficiency advantages, conventional heat curing remains superior in clinical durability. These results provide clinicians with evidence‐based guidance on balancing laboratory efficiency with long‐term patient outcomes. This means that dentists can apply straightforward surface modifications to achieve longer‐lasting dentures without additional cost or specialized equipment, thus directly benefiting patient outcomes.

This process involves creating small indentations or grooves on the surface of the artificial teeth. These indentations help to enhance the bond between the denture base resin and the teeth, ensuring a stronger and more durable denture in agreement with Cunningham and Benington [30]. The research findings, therefore, align with these findings. A limitation of this study is that only SBS was evaluated. Other clinically relevant mechanical properties, such as aging resistance, water sorption, and fatigue failure, were not assessed. Future studies incorporating these tests will provide a more comprehensive understanding of denture base–tooth adhesion and its long‐term clinical behavior. These additional tests will be essential in future research to provide a more comprehensive understanding of denture base–tooth adhesion and to enrich the clinical relevance of laboratory findings.

## 5. Conclusions

The current experimental setup allows for the following deductions to be made:

1. The traditional heat‐polymerized acrylic resin outperformed the microwave‐polymerized acrylic resin in terms of overall binding strength to the denture teeth tested.
2. The vertical retention grooves carved into the tooth’s ridge lap surface, which promote retention to the acrylic resin denture foundation, strengthen the link between the denture teeth and denture base resin.
3. In both conventional and microwave curing processes, grooves with thinner developed a stronger shear bond to denture teeth. This novel finding provides direct clinical insight: combining mechanical and solvent treatments can meaningfully enhance denture durability and reduce clinical repair frequency, offering a cost‐effective solution that can be readily applied in daily prosthodontic practice.
4. In all untreated groups, particularly those that have been microwave‐cured, the mechanisms of failure are often sticky. The state of the teeth’s bonding surface is indicated by a pronounced cohesive indication.
5. A tooth surface treated with acetone increased the SBS, albeit not as much as a thinner surface.

Beyond laboratory measurements, this work highlights practical strategies to strengthen the acrylic tooth–denture base interface. Incorporating grooves with solvent conditioning, particularly thinner ones, can be readily adopted in clinical workflows without added cost or specialized equipment, thereby offering immediate translational impact.

## Ethics Statement

It is essential to remember that ethical considerations must always be taken into account when conducting research, regardless of the topic. The acquisition of ethical permission guarantees that the research follows specific tenets and directives that safeguard the welfare and entitlements of subjects or participants. Researchers should prioritize ethical practices in their research pursuits and be aware of the potential influence that their work may have on living beings, even though this particular study may not have required ethical approval.

## Disclosure

All AI‐assisted text was carefully reviewed, verified, and edited by the author to ensure accuracy and integrity of the content.

## Conflicts of Interest

The author declares no conflicts of interest.

## Author Contributions

The author contributed to the text and was helpful throughout the research process. The author took a proactive role in the design of the study, selecting the best research techniques, and ensured the integrity and authenticity of the information gathered. The author also worked closely to examine the data and derive insights from the results.

## Funding

The author received no funding for this work.

## Data Availability

The author elects to not share data.

## References

[1] Broers D. L. M. , Dubois L. , de Lange J. , Su N. , and de Jongh A. , Reasons for Tooth Removal in Adults: A Systematic Review, International Dental Journal. (2022) 72, no. 1, 52–57, 10.1016/j.identj.2021.01.011. 33648772 PMC9275356

[2] Asli H. N. , Rahimabadi S. , Belyani N. , Asli M. N. , and Falahchai M. , Effect of Different Mechanical Surface Treatments on Flexural Strength of Repaired Denture Base, Journal of Oral Research. (2022) 11, no. 6, 1–10, 10.17126/joralres.2022.066.

[3] Prpić V. , Schauperl Z. , Glavina D. , Ćatić A. , and Čimić S. , Comparison of Shear Bond Strengths of Different Types of Denture Teeth to Different Denture Base Resins, The Journal of Advanced Prosthodontics. (2020) 12, no. 6, 376–382, 10.4047/jap.2020.12.6.376. 33489022 PMC7790604

[4] Wongsa P. , Chaijareenont P. , and Angkasith P. , Effect of Surface Treatment Methods on Shear Bond Strength Between Polyamide and PMMA Denture Base Resins, Journal of International Dental and Medical Research. (2023) 16, no. 3.

[5] Pero A. C. , Scavassin P. M. , Nunes É.M. , Policastro V. B. , Giro G. , and Compagnoni M. A. , Bond Strength of Artificial Teeth Attached to a Microwave-Polymerized Denture Base Resin After Immersion in Disinfectant Solutions, Journal of Prosthodontics. (2016) 25, no. 7, 576–579, 10.1111/jopr.12354, 2-s2.0-84951188497. 26489039

[6] Sadar L. and Dhume S. , Comparative Evaluation of Shear Compressive Bond Strength Between Cross-Linked Acrylic Resin Denture Base and Cross-linked Acrylic Resin Teeth With Different Modifications of Their Ridge Lap Surfaces, The Journal of Contemporary Dental Practice. (2013) 14, no. 5, 898–903, 10.5005/jp-journals-10024-1423, 2-s2.0-84903195245. 24685795

[7] Patil S. B. , Naveen B. H. , and Patil N. P. , Bonding Acrylic Teeth to Acrylic Resin Denture Bases: A Review, Gerodontology. (2006) 23, no. 3, 131–139, 10.1111/j.1741-2358.2006.00129.x, 2-s2.0-39049174384. 16919093

[8] Darbar U. R. , Huggett R. , and Harrison A. , Denture Fracture—A Survey, British Dental Journal. (1994) 176, no. 9, 342–345, 10.1038/sj.bdj.4808449, 2-s2.0-0028767399. 8024869

[9] Page M. J. , McKenzie J. E. , and Bossuyt P. M. , et al.The PRISMA. 2020 Statement: An Updated Guideline for Reporting Systematic Reviews, BMJ. (2021) 372, no. n71. 10.1136/bmj.n71 PMC8005924 33782057

[10] Choi J. J. E. , Uy C. E. , and Plaksina P. , et al.Bond Strength of Denture Teeth to Heat-Cured, CAD/CAM and 3D Printed Denture Acrylics, Journal of Prosthodontics. (2020) 29, no. 5, 415–421, 10.1111/jopr.13125. 31697004

[11] Sayed M. E. , Lunkad H. , and Fageeh I. , et al.Comparative Evaluation of Compressive Bond Strength Between Acrylic Denture Base and Teeth With Various Combinations of Mechanical and Chemical Treatments, Coatings. (2021) 11, no. 12, 10.3390/coatings11121527, 1527.

[12] Cervino G. , Cicciù M. , Herford A. S. , Germanà A. , and Fiorillo L. , Biological and Chemo-Physical Features of Denture Resins, Materials. (2020) 13, no. 15, 10.3390/ma13153350, 3350. 32731445 PMC7435594

[13] Costa M. , Neves S. , Carvalho J. , Arantes-Oliveira S. , and Félix S. , In Vitro Comparative Study of Microhardness and Flexural Strength of Acrylic Resins Used in Removable Dentures, Medical Sciences Forum. (2021) 5, 10.3390/msf2021005045, 45.

[14] Raszewski Z. , Toporowska A. N. , Nowakowska D. , and Więckiewicz W. , Update on Acrylic Resins Used in Dentistry, Mini-Reviews in Medicinal Chemistry. (2021) 21, no. 15, 2130–2137, 10.2174/1389557521666210226151214. 33634758

[15] Ramakrishnan H. , Sree Varun M. , Krishnan C. S. , Jayakrishnakumar S. , and Azhagarasan N. S. , Comparative Evaluation of the Effect of Different Surface Treatments on Shear Bond Strength Between Silicone Soft Liner and Denture Base Resin — a Three-Dimensional Study, IOSR Journal of Dental and Medical Sciences. (2019) 18, no. 11, 24–34.

[16] Prpić V. , Ćatić A. , Kraljević Šimunković S. , Bergman L. , and Ćimić S. , The Shear Bond Strength Between Milled Denture Base Materials and Artificial Teeth: A Systematic Review, Dentistry Journal. (2023) 11, no. 3, 10.3390/dj11030066, 66. 36975564 PMC10046986

[17] Helal M. A. , Baraka Y. , Sanad M. E. , Ali A. , and Osman E. , Comparative Effect of Different Surface Treatments on the Shear Bond Strength of Two Types of Artificial Teeth Bonded To Two Types of Denture Base Resins, Journal of Prosthodontics. (2022) 31, no. 5, 427–433, 10.1111/jopr.13425. 34480386

[18] Naji G. A..-H. , Influence of Various Chemical Surface Treatments, Repair Materials, and Techniques on Transverse Strength of Thermoplastic Nylon Denture Base, International Journal of Dentistry. (2020) 2020, 10, 10.1155/2020/8432143, 8432143. 32963534 PMC7499321

[19] Theane H. P. , Cuew C. L. , and Goh K. I. , Shear Bond Strength of Denture Teeth to the Base: A Comparative Study, Quintessence International. (1996) 27, no. 6, 425–428. 8941837

[20] Takahashi Y. , Chai J. , Takahashi T. , and Habu T. , Bond Strength of Denture Teeth to Denture Base Resins, The International journal of prosthodontics. (2000) 13, no. 1, 59–65. 11203611

[21] Schneider R. I. , Curtis E. R. , and Clancy J. M. S. , Tensile Bond Strength of Acrylic Resin Denture Teeth to a Microwave or Heat Processed Denture Base, The Journal of Prosthetic Dentistry. (2002) 88, no. 2, 145–150, 10.1067/mpr.2002.127898, 2-s2.0-0036673537. 12397241

[22] Al-Dwairi Z. N. , Tahboub K. Y. , Baba N. Z. , Goodacre C. J. , and Özcan M. , A Comparison of the Surface Properties of CAD/CAM and Conventional Polymethylmethacrylate (PMMA), Journal of Prosthodontics. (2019) 28, no. 4, 452–457, 10.1111/jopr.13033, 2-s2.0-85061906470. 30730086

[23] Batisse C. and Nicolas E. , Comparison of CAD/CAM and Conventional Denture Base Resins: A Systematic Review, Applied Sciences. (2021) 11, no. 13, 10.3390/app11135990, 5990.

[24] Al-Somaiday H. M. , Rafeeq A. K. , and Al-Samaray M. E. , Effect of Different Surface Modifications of Acrylic Teeth and Thermocycling on Shear Bond Strength to Polycarbonate Denture Base Material, International Journal of Biomaterials. (2022) 2022, 1–6, 10.1155/2022/9855836, 9855836. PMC8847005 35178094

[25] Abu-Anzeh R. H. , Evaluation of Tensile Bond Strength of Tooth Denture Base Resin as a Function of Different Surface Treatments and Processing Regimes, 2003, University of Baghdad, MSc Thesis.

[26] Hariharan S. , Bhardwaj V. , Bala I. , Sitterberg J. , Bakowsky U. , and Ravi Kumar M. N. V. , Design of Estradiol Loaded PLGA Nanoparticulate Formulations: A Potential Oral Delivery System for Hormone Therapy:,, Pharmaceutical Research. (2006) 23, no. 1, 184–195, 10.1007/s11095-005-8418-y, 2-s2.0-32244438967. 16267632

[27] Khanna A. , Bhatnagar V. M. , Karani J. T. , Madria K. , and Mistry S. , A Comparative Evaluation of Shear Bond Strength Between Two Commercially Available Heat-Cured Resilient Liners and Denture Base Resin With Different Surface Treatments, Journal of Clinical and Diagnostic Research. (2015) 9, no. 5, ZC30–ZC34, 10.7860/JCDR/2015/11504.5892, 2-s2.0-84928995617. PMC4484150 26155558

[28] Vergani C. E. , Seiko M. Y. , Pavarina A. C. , Mangueira D. F. , and Giampaolo E. T. , Effect of Surface Treatment on Bond Strength Between Composite Resin and Acrylic Resin Denture Teeth, International Journal of Prosthodontics. (2000) 13, no. 5, 383–386. 11203658

[29] Al-Ani M. J. , The Effect of Different Surface Treatments on the Transverse Strength and Deflection of Repaired Acrylic Specimens, 2000, University of Baghdad, MSc Thesis.

[30] Cunningham J. L. and Benington I. C. , A New Technique for Determining the Denture Tooth Bond, Journal of Oral Rehabilitation. (1996) 23, no. 3, 202–209, 10.1111/j.1365-2842.1996.tb01234.x, 2-s2.0-0030097655. 8667127

